# Diagnostic Performance of PI-RADS v2, Proposed Adjusted PI-RADS v2 and Biparametric Magnetic Resonance Imaging for Prostate Cancer Detection: A Preliminary Study

**DOI:** 10.3390/curroncol28030169

**Published:** 2021-05-12

**Authors:** Guan-Hui Cai, Qi-Hua Yang, Wen-Bo Chen, Qing-Yu Liu, Yu-Rong Zeng, Yu-Jing Zeng

**Affiliations:** 1 Radiology Department, Huizhou Municipal Central Hospital, Huizhou 516001, China; likev520@163.com (G.-H.C.); wbchen5@163.com (W.-B.C.); zeng_yurong@163.com (Y.-R.Z.); zfx912000@163.com (Y.-J.Z.); 2 Radiology Department, Sun Yat-sen Memorial Hospital, Sun Yat-sen University, Guangzhou 510120, China; yangqh2@mail.sysu.edu.cn; 3 The Seventh Affiliated Hospital, Sun Yat-sen University, 628 Zhenyuan Road, Xinhu Street, Guangming New District, Shenzhen 518107, China

**Keywords:** prostate neoplasm, prostate cancer, multiparametric MRI, PI-RADS, Gleason score

## Abstract

Purpose: To evaluate the diagnostic performance of PI-RADS v2, proposed adjustments to PI-RADS v2 (PA PI-RADS v2) and biparametric magnetic resonance imaging (MRI) for prostate cancer detection. Methods: A retrospective cohort of 224 patients with suspected prostate cancer was included from January 2016 to November 2018. All the patients underwent a multi-parametric MR scan before biopsy. Two radiologists independently evaluated the MR examinations using PI-RADS v2, PA PI-RADS v2, and a biparametric MRI protocol, respectively. Receiver operating characteristic (ROC) curves for the three different protocols were drawn. Results: In total, 90 out of 224 cases (40.18%) were pathologically diagnosed as prostate cancer. The area under the ROC curves (AUC) for diagnosing prostate cancers by biparametric MRI, PI-RADS v2, and PA PI-RADS v2 were 0.938, 0.935, and 0.934, respectively. For cancers in the peripheral zone (PZ), the diagnostic sensitivity was 97.1% for PI-RADS v2/PA PI-RADS v2 and 96.2% for biparametric MRI. Moreover, the specificity was 84.0% for biparametric MRI and 58.0% for PI-RADS v2/PA PI-RADS v2. For cancers in the transition zone (TZ), the diagnostic sensitivity was 93.4% for PA PI-RADS v2 and 88.2% for biparametric MRI/PI-RADS v2. Furthermore, the specificity was 95.4% for biparametric MRI/PI-RADS v2 and 78.0% for PA PI-RADS v2. Conclusions: The overall diagnostic performance of the three protocols showed minimal differences. For lesions assessed as being category 3 using the biparametric MRI protocol, PI-RADS v2, or PA PI-RADS v2, it was thought prostate cancer detection could be improved. Attention should be paid to false positive results when PI-RADS v2 or PA PI-RADS v2 are used.

## 1. Introduction

Prostate cancer is the second most frequent cancer in males worldwide [1], and its morbidity has risen over recent decades as the global population has continued to age and lifestyles have continued to change [2]. In 2015, prostate cancer ranked sixth among all male malignant neoplasms in China [3]. To date, the multi-parametric magnetic resonance imaging (mp-MRI) technique is the best and most accurate imaging method for prostate cancer detection, localization, and local staging [4,5]. However, variations in scan protocols, interpretation, and reporting of prostate MRI exams in different institutes have led to inconsistency in MRI reports, and thus hindered its clinical application [6].

As a product of international collaboration among the American College of Radiology, ESUR, and AdMetech Foundation, the second version of PI-RADS (PI-RADS v2) was released [6] and used as the guideline for prostate cancer MR diagnosis and reporting. The second version clarified the assessment criteria for different MRI sequences for different zones of the prostate. For the peripheral zone (PZ), diffusion weighted imaging (DWI) is the primary determining sequence (dominant technique), while for the transition zone (TZ), T2WI is the dominant technique. Combination assessment with other sequences is needed for PI-RADS category 3 lesions, which also clarifies the classification standard that correlates with the presence of a clinically significant prostate cancer.

Although PI-RADS v2 has been used worldwide, its false negative rate could still be as high as 14% [7]. In 2016, Rosenkrantz et al. [8] proposed several adjustments to PI-RADS v2 based on their research of 343 cases of clinically suspected prostate cancer. The proposed adjusted PI-RADS v2 (PA PI-RADS v2) focused on how to upgrade lesions of PI-RADS category 3 and 4 using DWI or dynamic contrast enhanced-MRI (DCE-MRI), both of which may have better performance in detecting a Gleason score (GS) ≥ 7 tumor according to Rosenkrantz’s results [8]. Nonetheless, more clinical data support is needed.

A biparametric MRI assessment (T2WI + DWI) is also an important method for prostate cancer screening [9,10,11,12]. It is less time consuming and does not require administration of a contrast agent. In order to detect clinically significant prostate cancer, its diagnostic accuracy and sensitivity is comparable to that of full mpMRI protocols [9].

There are both advantages and disadvantages for the three protocols mentioned above (PI-RADS v2, PA PI-RADS v2, and bpMRI). However, to the best of our knowledge, there are few studies that compare the diagnostic performance of such protocols (12–15). Our aim in this study was to compare the diagnostic performance of PI-RADS v2, PA PI-RADS v2, and biparametric MRI in prostate cancer detection.

## 2. Materials and Methods

### 2.1. Patients

**Figure 1:**
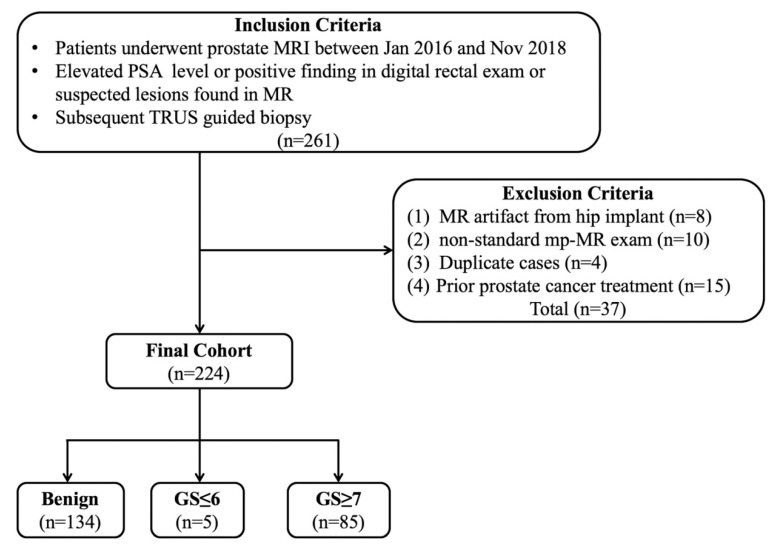
Flowchart of the study population. Note: TRUS—transrectal US; PSA—prostate-specific antigen.

This retrospective study was approved by our Institutional Review Board (protocol no.LLBA201936A), and written informed consent was waived. In total, 261 cases of prostate MRI performed between January 2016 and November 2018 were retrospectively reviewed. The inclusion criteria were as follows: (1) patients with elevated prostate-specific antigen (PSA) levels, positive in digital rectal examination or with suspected lesions found in MRI and (2) transrectal ultrasound guided fine needle biopsy was performed after the MRI scan. Moreover, the exclusion criteria were as follows: (1) a marked artifact induced by a hip implant; (2) the PI-RADS assessment could not be finished due to a non-standard mpMRI scan; (3) duplicate cases; (4) prior treatment including endocrine therapy, cryotherapy, and radiotherapy (Figure 1).

### 2.2. MR Imaging

All the prostate MR examinations were performed on a clinical Philips 1.5 Tesla scanner (Multiva, Philips Healthcare, Best, The Netherlands) by using a 16-channel phased-array coil. Pulse sequences included axial T1-weighted imaging (T1WI), turbo spin-echo T2-weighted imaging (T2WI), single-shot echo-planar DWI, and DCE-MRI (Table 1). B values of 0, 800, and 1500 s/mm^2^ were used for DWI. For DCE-MRI, 0.1 mmol/kg Gd-GDTA (Magnevist, Bayer Healthcare, Leverkusen, Germany) was administered at a rate of 2.5 mL/s via a power injector. The whole DCE scan lasted for about 320 s for 16 phases with a temporal resolution of 6.3 s per phase.

### 2.3. Imaging Assessment

All the MR examinations and prostate biopsy results were initially reviewed by a senior radiologist with 20 years of experience in prostate MR imaging. Suspicious lesions were digitally marked with elliptical regions of interest (ROI) as targeted lesions to ensure that both readers would evaluate the same lesion. Before the evaluation, patient images were randomized, anonymized, and a timeframe of 3 weeks was maintained to minimize reporting bias. Subsequently, two radiologists with more than 5 years of experience in abdomen MR imaging independently evaluated MR examinations of previously noted targeted lesions. A score of 1–5 was assigned according to PI-RADS v2, PA PI-RADS v2, and biparametric MRI protocols. Next, PI-RADS categories 4 and 5 were set as malignant [6,8,9]. Adjustments of PA PI-RADS v2 upgraded category 3 to 4 if the DWI score was 4 or if the modified DCE score was positive in TZ. Furthermore, it upgraded category 4 to 5 when the lesion size was 10–14 mm in PZ or TZ [8]. These two radiologists were blinded to the pathological results and the senior radiologist trained them to use the system. All pulse sequences were reviewed in a single session. The readers assigned each lesion a score on a scale of 1–5 for both T2W imaging and DWI, while DCE was classified as either positive or negative according to the three protocols.

### 2.4. Pathology Reference

Transrectal ultrasound guided prostate biopsy was performed by urologists in all the patients within two weeks after an MR scan. All the suspected lesions found on mpMR were located according to the 6-zone method. Moreover, the ultrasound guided 12-core biopsy was performed afterwards. Records included the biopsy site and Gleason score.

### 2.5. Statistical Analysis

Statistical analysis was performed with the SPSS software (version 22.0, IBM, Armonk, NY, USA) and MedCalc Statistical software (version 15.2.2, MedCalc Software bvba, Ostend, Belgium; 2015).

An inter-reader agreement was assessed using Kappa statistics, and the degree of agreement was classified as follows: κ > 0.8, perfect; 0.8 ≥ κ > 0.6, good; 0.6 ≥ κ > 0.4, moderate; 0.4 ≥ κ > 0.2, fair; 0.2 ≥ κ > 0, poor. The receiver operating characteristic (ROC) curve and decision curve analysis (DCA) of PI-RADS v2, PA PI-RADS v2, and biparametric MRI were drawn for diagnosing prostate cancer (biopsy pathological result was used as a reference). The diagnostic sensitivity, specificity, accuracy, positive predictive value (PPV), negative predictive value (NPV), and area under the ROC curve (AUC) were calculated, and the Z test was used to compare the AUC. A *t* test for independent samples was used to compare the PSA level of prostate cancer and benign lesions, while a Kolmogorov–Smirnov was used as normality test. *p* < 0.05 was defined as statistically significant.

## 3. Results

**Figure 2:**
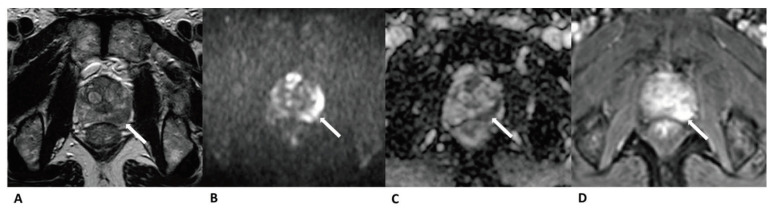
Case of prostate cancer in PZ. Male, 64 years old, prostate cancer with its longest diameter being >1.5 cm. T2WI showed a hypointense nodule in the left PZ (arrow) (**A**). The lesion was hyperintense on DWI (b = 1500 s/mm^2^) (arrow) and hypointense on the ADC images (**B**,**C**). Positive enhancement was seen on the DCE (arrow) (**D**). This lesion was finally assessed as a PI-RADS category 5, indicating that it had a high cancer risk according to PI-RADS v2, PA PI-RADS v2, and biparametric MRI protocols.

**Figure 3:**
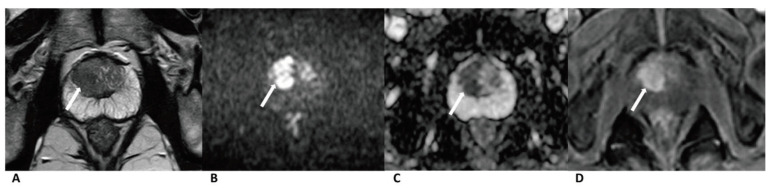
Case of prostate cancer in TZ. Male, 63 years old, prostate cancer with its longest diameter being >1.5cm. T2WI showed a hypointense nodule (arrow) in the right TZ (**A**). The lesion was hyperintense on DWI (b = 1500 s/mm^2^) (arrow) and hypointense on ADC images (**B**,**C**). Positive enhancement was seen on the DCE (arrow) (**D**). The lesion was finally assessed as a PI-RADS category 5, indicating that it had a high cancer risk according to PI-RADS v2, PA PI-RADS v2, and biparametric MRI protocols.

**Figure 4:**
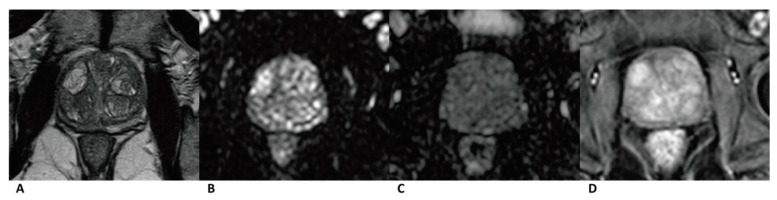
Case of benign prostate hyperplasia. Male, 64 years old, benign prostate hyperplasia. T2WI showed multiple slightly hypo/hyper-intense encapsulated nodules in TZ (**A**). The lesion was isointense on the DWI and ADC map (**B**,**C**). A diffuse popcorn-like enhancement was seen on the DCE and was assessed as negative (**D**). It was finally assessed as a category 2, indicating that it had a low cancer risk according to PI-RADS v2, pa PI-RADS v2, and biparametric MRI protocols.

A total of 224 patients (mean age: 69 ± 7.88 years, median age: 69 years, IQR: 12 years) were included. Clinical data and Gleason score results are listed in Table 2. In total, 90 cases were diagnosed as clinically significant prostate cancer, whereas 52 were diagnosed PZ and 38 in TZ. Further, 134 cases were diagnosed as benign lesions, whereas 25 were diagnosed PZ and 109 in TZ (Figure 2, Figure 3 and Figure 4).

**Figure 5:**
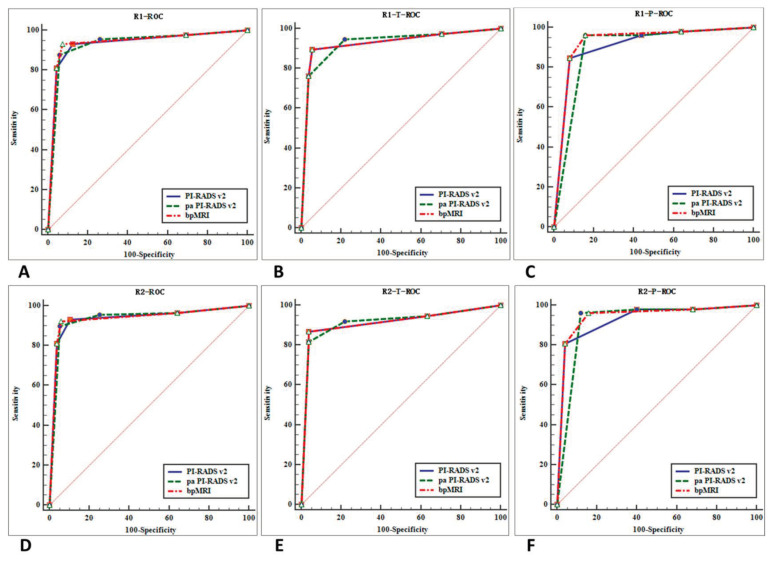
ROC curves for the diagnostic performance of two reviewers who used the three protocols. ROC curves of reviewer 1 for the diagnosis of prostate cancer (TZ + PZ) (**A**), prostate cancer in TZ (**B**), and PZ (**C**). ROC curves of reviewer 2 for the diagnosis of prostate cancer (TZ + PZ) (**D**), prostate cancer in TZ (**E**), and PZ (**F**).

**Figure 6:**
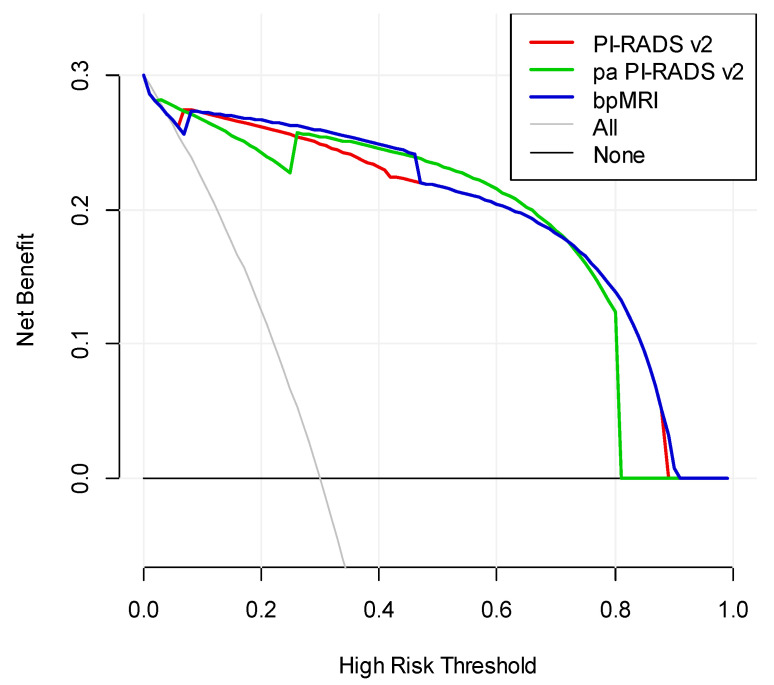
DCA for the diagnostic performance of the three protocols.

We drew ROC curves and DCA for the three protocols for diagnosing prostate cancer (Figure 5 and Figure 6) and AUCs. Our findings are as follows: (1) for all 224 cases (TZ + PZ), AUC of biparametric MRI (0.938) > PI-RADS v2 (0.935) > PA PI-RADS v2 (0.934); (2) for lesions in PZ (*n* = 77), AUC of biparametric MRI (0.9365) > PI-RADS v2 (0.9200) > PA PI-RADS v2 (0.9090); (3) for lesions in TZ (*n* = 147), AUC of PA PI-RADS v2 (0.9225) > biparametric MRI (0.9205) = PI-RADS v2 (0.9205). The difference was not statistically significant (*p* > 0.05) for all of the AUC comparisons mentioned above.

As the three protocol are cross in DCA, it is hard to say which protocol is better. However, we can conclude from the DCA that when the high-risk threshold in the interval of 0.1 to 0.45, bpMRI is better, while pa PI-RADS v2 is better in the interval of 0.5 to 0.7. When the high-risk threshold is larger than 0.75, pa PI-RADS v2 is similar to PI-RADS v2 and better than bpMRI.

Among all the significant prostate cancers, biparametric MRI misdiagnosed 4 lesions as category 3 (1 in PZ and 3 in TZ). Two misdiagnosed lesions in TZ were correctly detected using PA PI-RADS v2. The one in PZ was correctly detected using both PI-RADS v2 and PA PI-RADS v2.

Among all clinically significant prostate cancers, malignant lesions (TZ + PZ) were evaluated as category 3 according to the primary determining sequence (DWI for PZ and T2WI for TZ). They were finally upgraded to category 4 after combining other sequences as follows: 3 (3.3%) for PI-RADS v2, 5 (5.6%) for PA PI-RADS v2, and 2 (2.2%) for biparametric MRI. For benign lesions (TZ + PZ) upgraded from category 3 to 4 after combining other sequences, the misdiagnosed ones were as follows: 5 (3.7%) for PI-RADS v2, 26 (19.4%) for PA PI-RADS v2, and 0 (0.0%) for biparametric MRI (Table 3).

According to the Landis and Koch standard, an inter-observer agreement showed a good match in PI-RADS v2 (κ = 0.64, *p* < 0.001) and biparametric MRI (κ = 0.63, *p* < 0.001). A moderate match was found in the PA PI-RADS v2 (κ = 0.571, *p* < 0.001) and DCE results (κ = 0.58, *p* < 0.001) (Table 4).

The diagnostic accuracy for prostate cancer (category ≥ 4) according to the three protocols (PI-RADS v2, PA PI-RADS v2, and biparametric MRI) via the two radiologists was 93.3% and 93.3%; 95.6% and 95.6%; and 93.3% and 92.2%, respectively. The diagnostic accuracy for prostate benign lesions (category ≤ 3) according to the three protocols proposed by the two radiologists was 87.3% and 89.2%; 73.9% and 74.6%; and 92.5% and 94.0%, respectively (Table 5).

Table 6 shows the diagnostic sensitivity, specificity, accuracy, NPV, and PPV for prostate cancer for TZ, PZ, and the whole prostate (TZ + PZ) of the three protocols. For lesions in PZ, the sensitivity was highest for PI-RADS v2 and PA PI-RADS v2 (97.12%), while the specificity and accuracy were the highest for biparametric MRI (84.00% and 92.21%, respectively). For lesions in TZ, the sensitivity was the highest for PA PI-RADS v2 (93.42%), while the specificity and accuracy were the highest in PI-RADS v2 and biparametric MRI (95.41% and 93.54%, respectively). For the whole (PZ + TZ), the sensitivity was the highest for PA PI-RADS v2 (95.56%), whereas the specificity and accuracy were highest in biparametric MRI (93.28% and 93.08%, respectively).

## 4. Discussion

PI-RADS v2 is a new MRI report system that aims to detect clinically significant prostate cancer (defined via pathology or histology as having a Gleason score of ≥7, volume of ≥0.5cm^3^, and/or extra prostate extension) [6]. Previous studies confirmed its role in detecting prostate cancers [13,14,15,16], but drawbacks were present [7,17].

For the PZ lesion, the assessment criteria were almost the same between the PI-RADS v2 and PA PI-RADS v2 protocols. Only the standards for upgrading PI-RADS from a category 4 to 5 were different, but this did not affect the detection of prostate cancer in PZ. For the TZ lesion, the assessment criteria were the same between the PA PI-RADS v2 and biparametric MRI protocol. Thus, our study mainly focused on comparing the diagnostic performances of (1) PI-RADS v2/PA PI-RADS v2 and biparametric MRI in PZ and (2) PI-RADS v2/biparametric MRI and PA PI-RADS v2 in TZ, and (3) the overall performance of the three protocols in TZ + PZ.

The inter-observer agreements of PI-RADS v2 and biparametric MRI were better than that PA PI-RADS v2, which was moderate. PA PI-RADS v2 revised the upgrading standard (for category 3 to 4 and category 4 to 5), which made the assessment more complicated and may be the main reason for the lower inter-observer agreement.

### 4.1. Diagnostic Performance Comparison in PZ

In this study, the diagnostic performance of biparametric MRI and PI-RADS v2/PA PI-RADS v2 were without statistical distinction in PZ lesions. The main difference between PI-RADS v2/PA PI-RADS v2 and biparametric MRI is that the latter does not take DCE results into consideration while the former criteria upgrades lesions from category 3 to 4 when the DCE results are positive [6,8]. In this study, only one case was correctly detected due to upgrading using PI-RADS v2/PA PI-RADS v2, and the detection rate only increased by 1.9%. In the meantime, however, five (PI-RADS v2) and seven (PA PI-RADS v2) cases of benign lesions were misdiagnosed as prostate cancer with the same protocol, which led to a 20.0% and 28.0% misdiagnosis rate, respectively. The false positivity in DCE can lead to over diagnosis, which should not be clinically neglected. The accuracy of PI-RADS v2/PA PI-RADS v2 was lower than biparametric MRI in PZ lesions. Vargas et al. found that DCE offered limited added value to T2WI + DWI-MR [18]. However, Greer et al. found that adding DCE to DWI scores in the PZ yielded meaningful improvements in prostate cancer detection (16%) [19]. To date, the diagnostic value of prostate DCE MRI is still controversial [20,21,22] and careful consideration is needed for its application in upgrading a category 3 lesion in PZ.

The specificity of PI-RADS v2/PA PI-RADS v2 was relatively low in this study, which may be due to the small sample size of benign lesions in PZ and false positive enhancement in benign lesions including prostatitis, hyperplasia, and intraepithelial neoplasia [23,24,25]. Although the inter-reader reproducibility of DCE in PZ was better in our research than in the literature [26], the rate of misdiagnosis due to DCE was still high, which implies that the two radiologists still had problems in differentiating benign and malignant lesions. Vargas found that the value of DCE is limited when considering side effects of the contrast medium, additional scan time, and cost [18]. Considering safety, scan time, and cost, biparametric MRI should be the first choice for prostate cancer screening. For clinical suspected malignant and biparametric MRI category 3 lesions in PZ, DCE could be applied to improve the detection rate.

### 4.2. Diagnostic Performance Comparison in TZ

For TZ lesions, the diagnostic performance between PA PI-RADS v2 and PI-RADS v2/biparametric MRI was comparable. The main difference between PI-RADS v2/biparametric MRI and PA PI-RADS v2 was that, for category 3 TZ lesions, the former two protocols upgraded lesions to category 4 only when they scored 5 in DWI, but the latter upgraded lesions to category 4 when they scored 4 in DWI or were positive in DCE [8]. Adjustments of upgrading standards can improve the detection rate of clinically significant cancer. Of the 38 prostate cancers in TZ, two cases were correctly detected by upgrading in PI-RADS v2/biparametric MRI, while four cases were correctly detected by upgrading in PA PI-RADS v2. Therefore, the sensitivity of PA PI-RADS v2 was higher than PI-RADS v2/biparametric MRI (93.42% vs. 88.16%).

In total, 19 (17.4%) out of 109 cases of benign lesions in TZ were misdiagnosed as cancer by upgrading the standard in PA PI-RADS v2. The specificity and accuracy of PA PI-RADS v2 (77.98% and 81.97%) for TZ lesions were lower than those of PI-RADS v2/biparametric MRI (95.41% and 93.54%). The high false positive rate could be attributed to the changes in upgrading the standard from category 3 to 4 in PA PI-RADS v2. Moreover, 14 misdiagnosed cases were upgraded by the positive DCE result, wherein the inter-reader agreement was only moderate (k = 0.408). The assessment based on the DCE image was, to some extent, subjective and controversial, which led to lower specificity and accuracy. Interpreting images of benign lesions is complicated, and it is sometimes hard to differentiate them from prostate cancer based on a DCE image [27]. Encapsulated swirled or popcorn-like enhancement patterns (Figure 3) caused by hypervascularity in prostate hyperplasia were defined as negative in PA PI-RADS v2 and could be used as a sign for diagnosing prostate hyperplasia [8]. However, this enhancement pattern was defined as positive in PI-RADS v2. Further studies are needed to testify for this standard in PA PI-RADS v2 and refine its details to help practitioners better understand the standard. No difference was found in the AUC of the ROC curve for the two protocols that diagnosed TZ lesions, although prior research found a higher specificity and accuracy for PI-RADS v2 [28,29,30,31]. Whether DCE could be used in microvascular anatomy and functional evaluation to improve the detection specificity and accuracy for TZ lesions needs further study [32,33,34,35].

### 4.3. Overall Diagnostic Performance of the Three Protocols

Our results showed minimal differences in the overall diagnostic performance of the three protocols. Although biparametric MRI removed DCE and evaluated lesions only based on T2WI and DWI, it had the highest specificity and accuracy and was the best at avoiding misdiagnoses for benign lesions such as cancer. The sensitivity was the highest in PA PI-RADS v2, but its specificity and accuracy were the worst, so it requires revision and improvement.

Prior research results were inconsistent regarding the diagnostic performance of the different protocols. Auer and Wang et al. [7,36] reported the diagnostic performance of PI-RADS v2 with AUCs of 0.878 and 0.900, respectively. Schimmoller and Hoeks et al. [22,37] reported that of biparametric MRI with AUCs of 0.762 and 0.81, respectively. Kuhl et al. [9] found the diagnostic accuracy of biparametric MRI comparable to that of PI-RADS v2 (89.10% vs. 87.20%), which was similar to our results.

In our research, after the combined evaluation with DCE results, 3 of the 4 misdiagnosed cases were correctly detected by either PA PI-RADS v2 or PI-RADS v2. Thus, for lesions of category 3 in biparametric MRI, the application of DCE via PA PI-RADS v2 or PI-RADS v2 protocols could improve cancer detection.

There were some limitations in this study. First, this is a retrospective study performed at a single institution. Second, we did not assess the performance of the three protocols in evaluating the prostate cancer staging. Third, the sample size for our evaluation was generally small. Further investigations by other centers/multi-centers with larger sample size are required to confirm our preliminary observations. Fourth, our study did not use 3.0T on prostate MRI scanning, which would be benefit the increasing signal-to-noise and resolution of images.

## 5. Conclusions

In conclusion, the diagnostic performance of PI-RADS v2, PA PI-RADS v2, and biparametric MRI resulted in good prostate cancer detection, with minimal significant differences among the methods. We recommend biparametric MRI for cancer screening because it saves time, does not need contrast media, and shows satisfactory diagnostic performance. For category 3 lesions found via the biparametric MRI protocol, DCE can improve the detection rate for clinically significant prostate cancer (for lesions in TZ, PA PI-RADS v2 can be used; for lesions in PZ, both PI-RADS v2 and PA PI-RADS v2 can be used). However, the specificity and accuracy will be lower when using DCE, especially for lesions in PZ. Further study is required with regard to how to improve detection. Moreover, further studies are needed to reduce the misdiagnosis of cancers.

## Figures and Tables

**Table 1:** MR scan parameters.

Sequence	FOV (mm)	ST (mm)	Gap (mm)	NSA	Matrix	TE (ms)	TR (ms)	Time	Phase
T1WI	200 × 200	3	0	1	224 × 224	1.7	4.9	1 min and 32 s	/
T2WI	180 × 180	3	0	3	256 × 256	85	4886	2 min and 54 s	/
DWI	200 × 200	3	0	8	100 × 100	86	4100	6 min and 19 s	/
DCE	240 × 240	3	0	1	224 × 224	1.5	4.9	5 min and 25 s	16

Note: FOV: field of view; ST: slice thickness; NSA: number of signal averaged; TR: repetition time; TE: echo time.

**Table 2:** Clinical pathologic data of 224 patients.

Variable	All Patients	Prostate Cancer	Benign Lesions	*p* Value
Numbers (*n*, %)	224	90 (40.81%)	134 (59.82%)	
Mean Age (years)	69.00 ± 7.88	70.62 ± 7.86	67.90 ± 7.73	0.476
Median Age (years)	69	71	68	
IQR (years)	12	12	11	
Median PSA Level (ng/mL) *	14.55 (0.68–616.80)	55.77 (1.05–616.80)	10.58 (0.68–101.00)	0.000
Location of Lesion				
PZ (n, %)	77 (34.38%)	52 (57.78%)	25 (18.66%)	0.000
TZ (n, %)	147 (65.62%)	38 (42.22%)	109 (81.34%)	0.000
Gleason Score (n, %) †				
≤3 + 3	5 (5.56%)			
3 + 4	10 (11.11%)			
4 + 3	21 (23.33%)			
≥4 + 4	54 (60.00%)			

Note: * Prostate specific antigen (PSA) level of prostate cancer patients was significantly higher than that in benign lesions patients (*p* < 0.05). † There were five cases with GS ≤ 3 + 3 in this study, volumes of these five lesions were all larger than 0.5 cm^3^ and could be defined as a significant cancer.

**Table 3:** Lesions of category 3 (according to the primary determining sequence) upgraded to category 4 after being combined with other sequences.

Protocol	TZ		PZ		TZ + PZ	
	PC	BH	PC	BH	PC	BH
PI-RADS v2	2/38 (5.3%)	0/109 (0.0%)	1/52 (1.9%)	5/25 (20.0%)	3/90 (3.3%)	5/134 (3.7%)
PA PI-RADS v2	4/38 (10.5%)	19/109 (17.4%)	1/52 (1.9%)	7/25 (28.0%)	5/90 (5.6%)	26/134 (19.4%)
Biparametric MRI	2/38 (5.3%)	0/109 (0.0%)	0/52 (0.0%)	0/25 (0.0%)	2/90 (2.2%)	0/134 (0.0%)

Note: PZ—peripheral zone; TZ—transition zone; PC—prostate cancer; BH—prostate hyperplasia.

**Table 4:** The values of Kappa of inter-observer agreement.

Protocol	Values of Kappa	Level of Agreement	% of Reliable Data	*p* Value
PI-RADS v2	0.64	good match	100%	<0.001
PA PI-RADS v2	0.571	moderate match	100%	<0.001
biparametric MRI	0.63	good match	100%	<0.001
DCE	0.58	moderate match	100%	<0.001

Note: the degree of agreement was classified as follows: κ > 0.8, perfect; 0.8 ≥ κ > 0.6, good; 0.6 ≥ κ > 0.4, moderate; 0.4 ≥ κ > 0.2, fair; 0.2 ≥ κ > 0, poor.

**Table 5:** PI-RADS assessments of the three protocols by the two radiologists.

	All Patient		Prostate Cancer		Benign Lesions	
	R1 *	R2 *	R1	R2	R1	R2
PI-RADS v2, n (%)						
1	0 (0)	0 (0)	0 (0)	0 (0)	0 (0)	0 (0)
2	45 (20.1)	51 (22.8)	2 (2.2)	3 (3.3)	43 (32.1)	48 (35.8)
3	78 (34.8)	75 (33.5)	4 (4.4)	3 (3.3)	74 (55.2)	72 (53.4)
4	22 (9.8)	20 (8.9)	11 (12.2)	11 (12.2)	11 (8.2)	9 (6.7)
5	79 (35.3)	78 (34.8)	73 (81.1)	73 (81.1)	6 (4.5)	5 (3.7)
PA PI-RADS v2, n (%)						
1	0 (0)	0 (0)	0 (0)	0 (0)	0 (0)	0 (0)
2	45 (20.1)	51 (22.8)	2 (2.2)	3 (3.3)	43 (32.1)	48 (35.8)
3	58 (25.9)	53 (23.7)	2 (2.2)	1 (1.1)	56 (41.8)	52 (38.8)
4	34 (15.2)	32 (14.3)	7 (7.8)	5 (5.6)	27 (20.1)	27 (20.1)
5	87 (38.8)	88 (39.3)	79 (87.8)	81 (90.0)	8 (6.0)	7 (5.2)
Biparametric MRI, n (%)						
1	0 (0)	0 (0)	0 (0)	0 (0)	0 (0)	0 (0)
2	45 (20.1)	51 (22.8)	2 (2.2)	3 (3.3)	43 (32.1)	48 (35.8)
3	85 (37.9)	82 (36.6)	4 (4.4)	4 (4.5)	81 (60.4)	78 (58.2)
4	15 (6.7)	13 (5.8)	11 (12.2)	10 (11.1)	4 (3.0)	3 (2.2)
5	79 (35.3)	78 (34.8)	73 (81.1)	73 (81.1)	6 (4.5)	5 (3.7)

Note: * R1—radiologist 1; R2—radiologist 2.

**Table 6:** Diagnostic performance of PI-RADS v2, PA PI-RADS v2, and biparametric MRI protocols for prostate cancer.

Variable	PZ	TZ	PZ + TZ	GS 3 + 4	GS 4 + 3
PI-RADS v2					
sensitivity	97.12%	88.16%	93.33%	90.00%	90.48%
specificity	58.00%	95.41%	88.43%	89.55%	89.55%
accuracy	84.42%	93.54%	90.40%	89.58%	89.68%
PPV	82.79%	87.09%	84.44%	39.13%	57.58%
NPV	90.63%	95.86%	95.18%	99.17%	98.36%
PA PI-RADS v2					
sensitivity	97.12%	93.42%	95.56%	100.00%	90.48%
specificity	58.00%	77.98%	74.25%	74.63%	74.63%
accuracy	84.42%	81.97%	82.81%	76.39%	76.77%
PPV	82.79%	59.66%	71.37%	22.73%	35.85%
NPV	90.63%	97.15%	96.14%	100.00%	98.04%
Biparametric MRI					
sensitivity	96.15%	88.16%	92.78%	90.00%	85.71%
specificity	84.00%	95.41%	93.28%	94.03%	94.03%
accuracy	92.21%	93.54%	93.08%	93.75%	92.90%
PPV	92.59%	87.09%	90.29%	52.94%	69.23%
NPV	91.30%	95.86%	95.06%	99.21%	97.67%

Note: PZ—peripheral zone; TZ—transition zone; PPV—positive predictive value; NPV—negative predictive value; PZ + TZ—all cancers; GS 3 + 4—favorable intermediate cancers; GS 4 + 3—unfavorable intermediate cancers.

## Data Availability

Data is not available in the public domain due to privacy restrictions and therefore no links to publicly archived datasets are provided.
